# Sex‐specific adaptations to exercise training: Focus on autonomic modulation and oxidative stress in normotensive rats

**DOI:** 10.14814/phy2.70685

**Published:** 2025-12-02

**Authors:** Maycon Junior Ferreira, Guilherme Lemos Shimojo, Filipe Fernandes Stoyell‐Conti, Catarina Barboza, Michelle Sartori, Iris Callado Sanches, Maria Cláudia Irigoyen, Kátia De Angelis

**Affiliations:** ^1^ Exercise Physiology Laboratory Universidade Federal de São Paulo (UNIFESP) Sao Paulo Brazil; ^2^ Translational Physiology Laboratory Universidade Nove de Julho (UNINOVE) Sao Paulo Brazil; ^3^ Department of Surgery Leonard M. Miller School of Medicine, University of Miami Miami Florida USA; ^4^ Human Movement Laboratory Universidade São Judas Tadeu (USJT) Sao Paulo Brazil; ^5^ Hypertension Unit, Heart Institute (InCor) School of Medicine, Universidade de São Paulo (USP) Sao Paulo Brazil

**Keywords:** aerobic exercise training, heart rate variability, oxidative stress, sex differences, target organs

## Abstract

Sex‐related physiological differences influence health, disease, and responses to preventive interventions. We aimed to compare the effects of exercise training on cardiovascular autonomic modulation and oxidative stress in target organs, focusing on sex differences. Wistar rats were distributed into sedentary male (SM) and female (SF), and trained male (TM) and female (TF) groups. Arterial pressure was recorded intra‐arterially, and autonomic modulation was assessed. Rats underwent treadmill training (5 days/week, 8 weeks). Oxidative stress was evaluated in cardiac and renal tissues. There were sex‐related differences in anthropometric, functional, autonomic, and oxidative stress markers. In turn, aerobic exercise training led to significant enhancements in functional performance, cardiovascular autonomic control, and oxidative stress status. Notably, these benefits were more evident in females. Vascular sympathetic modulation correlated positively with renal lipid peroxidation (LPO). Cardiac LPO was lower in TM, SF, and TF (vs. SM). Only TF showed reduced renal LPO and improved cardiac redox balance. Trained females also demonstrated greater improvements in renal antioxidant capacity (TRAP: ~1.6 times), cardiac oxidized glutathione (~3.3 times) (vs. TM), and increased nitrite concentration (~2.1 times) (vs. SF). In conclusion, female rats exhibited greater improvements in cardiovascular autonomic modulation and oxidative profile in target organs in response to exercise training compared to male rats.

## 1 INTRODUCTION

The prevalence of cardiovascular diseases (CVDs) is higher in young and middle‐aged men than in women, suggesting that sex hormones may play a key role in the development of CVD (Vitale et al., 2009). Sex hormones exert metabolic, haemodynamic, and cardiac autonomic effects that may explain sex differences in the relevance of risk factors (Dart et al., 2002; Vitale et al., 2009). Clinical and experimental studies report that cardiovascular autonomic regulation also plays an important role in cardiac mortality (Kleiger et al., 1987; Schwartz et al., 1984). In this regard, a previous study showed that there are sex‐related differences in cardiac autonomic modulation (Venkata Pothineni et al., 2016). A cross‐sectional study of a cohort of healthy, normotensive males and females showed that changes in muscle sympathetic nerve activity burst frequency follow a nonlinear pattern at the population level, varying across different age groups (Keir et al., 2020). Furthermore, sex differences have been observed in the timing, direction, and magnitude of these age‐related changes in muscle sympathetic nerve activity (Keir et al., 2020). Furthermore, clinical evidence has shown that, compared to men, women tend to exhibit higher resting heart rate (HR), increased cardiac vagal tone, and reduced cardiac sympathetic activity (Joyner et al., 2015). Indeed, young women exhibit greater parasympathetic modulation and lower sympathetic modulation than men. However, sex‐related differences in parasympathetic regulation diminish after the age of 50, whereas sympathetic dominance disappears more slowly in men (Kuo et al., 1999). This coincides with menopause, when there is a marked reduction in female sex hormones. Together, these findings suggest that estrogen has a protective effect on the cardiac autonomic modulation response, while endogenous androgens have a detrimental effect on the development of CVDs.

Oxidative stress is another important mechanism involved in the pathophysiology of many diseases, such as hypertension, stroke and other CVDs (Kander et al., 2017; Liguori et al., 2018). Previous studies have suggested a link between sex and oxidative stress (Kander et al., 2017). In this regard, female Wistar rats exhibit higher antioxidant gene expression and lower oxidative stress levels than males (Borrás et al., 2003). Additionally, studies from our group have shown that autonomic dysfunctions, such as increased cardiac sympathetic modulation, are correlated with increased markers of oxidative stress (Bertagnolli et al., 2006; Irigoyen et al., 2005).

Modulation of the autonomic nervous system plays a key role in regulating inflammation by inhibiting the release of pro‐inflammatory cytokines (Bezerra et al., 2017; Kelly et al., 2022), and the pharmacological manipulation enhances both parasympathetic modulation and antioxidant defenses while reducing inflammation and oxidative stress (Bezerra et al., 2017). Along these lines, exercise triggers a cascade of physiological adaptations that enhance cardiovascular efficiency, improve metabolic health, and strengthen the body's resilience against inflammation and oxidative stress, thereby reducing the risk of chronic diseases (Fiuza‐Luces et al., 2013; Pedersen & Saltin, 2015). It is well known that acute exercise activates the sympathetic nervous system and generates reactive oxygen species (Christensen & Galbo, 1983; Powers et al., 2011). However, regular exercise training improves cardiovascular autonomic control upregulates the antioxidant profile in the heart and kidneys (Bayod et al., 2012; Shimojo et al., 2018), potentially conferring greater protection to target organs over time. In this regard, our group has demonstrated the benefits of cardiovascular and autonomic control parameters in healthy females (Sanches et al., 2009) and male rats (Farah et al., 2016) following a moderate‐intensity aerobic exercise training protocol.

To address this issue, we investigated sex differences and the effects of aerobic exercise training on cardiovascular autonomic regulation and oxidative stress profiles in the hearts and kidneys of healthy male and female rats. Given the critical role of the autonomic nervous system, in modulating inflammation and oxidative stress, it is important to evaluate autonomic function alongside oxidative stress markers in these target organs. The heart and kidneys are particularly vulnerable to autonomic dysregulation and redox imbalance, and oxidative stress is a reliable indicator of oxidative damage in these organs. Furthermore, we hypothesized that aerobic exercise training would induce benefits in these important mechanisms involved in the onset of CVDs in both sexes. We also tested the hypothesis that healthy females exhibit superior cardiovascular autonomic control and oxidative status in target organs than healthy males, and that exercise training positively modulates autonomic modulation and oxidative stress, thereby enhancing cardiorenal protection.

## 2 METHODS

Male and female Wistar rats (200–230 g, aged 3 months) were obtained from the São Judas Tadeu University Animal Facilities (São Paulo, SP, Brazil). The animals had free access to standard laboratory chow (Nuvital, Quimtia®, autoclave‐sterilizable) and water, and were housed in a room with a controlled temperature (22–25°C) and a controlled 12‐h light–dark cycle. The rats were randomly distributed into four groups (*n* = 6–8 rats/group): sedentary male (SM), trained male (TM), sedentary female (SF), and trained female (TF). All surgical procedures and protocols were approved by the Ethics Committee of São Judas Tadeu University (protocol no. 076/2004) and conducted in accordance with the National Guidelines for the Care and Use of Laboratory Animals. For the female groups, all in vivo evaluations and euthanasia were conducted during the non‐ovulatory phase of the estrous cycle. Based on the 3Rs principles in animal experimentation, we re‐used partial data (body weight, haemodynamic, arterial pressure (AP) variability (APV), and oxidative stress analyses) from some rats in the SM and SF groups previously published by our team (Ferreira et al., 2020) for this new study. In addition to these, we included a few new animals in the SM and SF groups to strengthen the dataset and allow for further analyses.

### 2.1 Aerobic exercise training

Aerobic exercise training was performed on a motor treadmill (Imbramed TK‐01, Brazil) at low‐to‐moderate intensity (approximately 50%–60% of the maximum running speed) for 1 h a day, 5 days a week, for 8 weeks. The speed was gradually increased from 0.3 to 1.0 km/h. All animals were acclimated to the experimental procedures (10 min per day at 0.3 km/h) for 5 days prior to the commencement of the exercise training protocol. After this adaptation period, the sedentary group only underwent exercise during the maximum treadmill test. To provide a similar environment and level of manipulation, sedentary animals were placed on a stationary treadmill three times a week. Sedentary and trained rats underwent a maximum treadmill test, as described in detail in a previous study (Rodrigues et al., 2007). Tests were performed at the beginning of the experiment and in the fourth and eighth weeks of the training protocol. The purpose of this was to determine exercise capacity and training intensity (Rodrigues et al., 2007).

### 2.2 Cardiovascular measurements

The day after the final exercise session, the rats were anesthetized with an intraperitoneal injection of ketamine (80 mg/kg) and xylazine (12 mg/kg). Two polyethylene‐tipped Tygon cannulas filled with heparinised saline were then implanted into the right carotid artery and jugular vein, respectively, to enable direct measurement of AP and drug administration. The free ends of the cannulas were tunneled subcutaneously and brought out at the top of the skull. To avoid detraining, haemodynamic measurements were taken from conscious, freely moving rats in their home cage at least 24 h after surgery, since no significant differences in AP values had been observed at that time (Bertagnolli et al., 2006; Kuo et al., 1999). The arterial cannula was connected to a transducer (Blood Pressure XDCR, Kent®, USA) and AP signals were recorded for 30 min using a microcomputer equipped with an analogue‐to‐digital converter (Windaq®, 2‐kHz, DATAQ Instruments®, USA). The recorded data were analyzed on a beat‐to‐beat basis to quantify changes in systolic (SAP), diastolic (DAP), and mean AP (MAP), as well as HR.

The standard deviation from the mean of three 5‐min time series for each animal was used to obtain pulse interval (PI) and SAP variabilities in the time domain. For the frequency domain analysis, cubic spline interpolation (250 Hz) and decimation were applied to the same time series of PI and SAP to equally space them in time after linear trend removal. Power spectral density was then obtained through Fast Fourier Transformation. The spectral power for the low‐frequency (LF: 0.20–0.75 Hz) and high‐frequency (HF: 0.75–4.0 Hz) bands was calculated by integrating the power spectrum density within each frequency band using a customized MATLAB 6.0 routine (Mathworks). The beat‐to‐beat values of the PI and SAP were then used to estimate the cardiac baroreflex sensitivity using spectral analysis and the alpha index in the low‐frequency band (0.20–0.75 Hz). Alpha index analysis evaluates short‐term changes in SAP and RR intervals. This method has been proposed to quantify causal events related to the baroreflex (Dias et al., 2020).

### 2.3 Oxidative stress evaluations

Following cardiovascular evaluation, the animals were submitted to euthanasia by decapitation. The heart (ventricles) and right kidney were promptly extracted, rinsed in saline and trimmed to eliminate excess fat and connective tissue. The tissues were then cut into small pieces, placed in an ice‐cold buffer, and homogenized using an Ultra‐Turrax blender at a ratio of 1 g of tissue to 5 mL of a solution containing 150 mmol/L KCl and 20 mmol/L phosphate buffer at pH 7.4. The homogenates were then centrifuged at 600*g* for 10 min at 2°C. Protein concentration was determined using the Lowry method with bovine serum albumin as the standard (Lowry et al., 1951).

#### 2.3.1 Lipid peroxidation

Lipid peroxidation (LPO) was measured by the tert‐butyl hydroperoxide‐initiated chemiluminescence (CL) assay, as previously described (Flecha et al., 1991). The CL assay was carried out with an LKB Rack Beta liquid scintillation spectrometer 1215 (LKB Producer AB) in the out‐of‐coincidence mode at room temperature (25°C–27°C). The supernatants were diluted in 140 mmol/L KCl and 20 mmol/L phosphate buffer, pH 7.4, and added to glass tubes, which were placed in scintillation vials; 3 mmol/L tert‐butylhydroperoxide was added, and CL was determined as the maximum level of emission.

#### 2.3.2 Total radical‐trapping antioxidant potential

Total radical‐trapping antioxidant potential (TRAP), which reflects the total antioxidant capacity in a homogenate, was measured by chemiluminescence using 2,2′‐azo‐bis(2‐amidinopropane) dihydrochloride (ABAP; A9941, Sigma‐Aldrich), a source of alkyl peroxyl free radicals, and luminol (A8511, Sigma‐Aldrich).

A mixture consisting of 20 mmol·L^−1^ luminol and 50 mmol·L^−1^ phosphate buffer (pH 7.4) was incubated to achieve a steady‐state luminescence resulting from free radical‐mediated luminol oxidation. A calibration curve was generated using different concentrations of Trolox (218940050, Acros), a water‐soluble analogue of vitamin E, ranging from 0.2 to 1 μmol·L^−1^ (Evelson et al., 2001). Luminescence was measured using a liquid scintillation counter operated in the out‐of‐coincidence mode.

#### 2.3.3 Antioxidant enzyme

Superoxide dismutase activity (SOD) was measured spectrophotometrically by rate inhibition of pyrogallol autoxidation at 420 nm (Hanschmann et al., 2013). Catalase activity (CAT) was measured by monitoring the decrease in H_2_O_2_ concentration at 240 nm (Aebi, 1984). Glutathione peroxidase activity (GPx) was determined by monitoring nicotinamide adenine dinucleotide phosphate (NADPH) oxidation spectrophotometrically at 340 nm (Flohé & Günzler, 1984).

#### 2.3.4 Determination of oxidized and reduced glutathione concentration

To determine oxidized (GSSG) and reduced glutathione (GSH) concentration, the cardiac tissue was deproteinized with 2 mol/L perchloric acid, centrifuged for 10 min at 1000*g*, and the supernatant was neutralized with 2 mol/L potassium hydroxide. The reaction medium contained 100 mmol/L phosphate buffer (pH 7.2), 2 mmol/L nicotinamide dinucleotide phosphate acid, 0.2 U/mL glutathione reductase, and 70 mmol/L 5,50′ dithiobis (2‐nitrobenzoic acid). To determine reduced glutathione, the supernatant was neutralized with 2 mol/L potassium hydroxide, to react with 70 mmol/L 5,50′ dithiobis (2‐nitro benzoic acid), and the absorbance values measured at 420 nm (Flohé & Günzler, 1984).

#### 2.3.5 Nitric oxide bioavailability

The bioavailability of nitric oxide was indirectly assessed by measuring systemic nitrite levels using the Griess reaction in 96‐well microplates. Briefly, 50 μL of Griess reagent was added to 50 μL aliquots of each sample and incubated at room temperature (Granger et al., 1999). The total nitrite concentration was quantified spectrophotometrically at 545 nm.

### 2.4 Statistical analysis

Data are presented as mean ± standard deviation. Distribution and homogeneity of variances were assessed using the Shapiro–Wilk and Levene's tests, respectively. A two‐way analysis of variance (ANOVA) was performed to evaluate the main effects of sex, exercise training, and the interaction between these factors. When statistically significant differences were found, appropriate post hoc tests were conducted to identify specific group comparisons. The Pearson correlation coefficient was used to investigate the relationship between parameters. The null hypothesis was rejected for values of p less than 0.05. All statistical analyses were performed using Jamovi software (version 2.7.6).

## 3 RESULTS

### 3.1 Metabolic evaluations

Body weight results are summarized in Table 1. A significant main effect of sex was observed for body weight (grams) at the beginning of the study (*p* < 0.001), with male rats exhibiting a higher body weight compared to females. These sex‐related differences were maintained at the end of the study, with males still exhibiting greater body weight (*p* < 0.001). Furthermore, a main effect of sex was observed for body weight gain (represented by delta) (*p* < 0.001) during the study, with males showing greater body weight gain compared to females. No main effect of exercise training on body weight was observed. However, a significant interaction effect between sex and exercise training was found for body weight at the beginning of the study (*p* = 0.028). Post hoc analysis revealed that body weight varied according to sex and exercise training, with sedentary and trained female rats showing lower body weight compared to sedentary and trained male rats.

**TABLE 1:** Body weight measurements and exercise test performance in the studied groups.

	Male		Female		Two‐way ANOVA		
	SM	TM	SF	TF	Sex effect	Exercise training effect	Interaction effect
Initial body weight, g	285 ± 16	297 ± 7	216 ± 14^#^ ^,^ ^†^	207 ± 15^#^ ^,^ ^†^	*p* < 0.001	*p* = 0.674	*p* = 0.028
Final body weight, g	382 ± 24	389 ± 27	265 ± 13	247 ± 14	*p* < 0.001	*p* = 0.441	*p* = 0.080
∆ body weight gain, g	97 ± 16	92 ± 25	49 ± 8	40 ± 16	*p* < 0.001	*p* = 0.215	*p* = 0.715
Initial exercise test, min	13.7 ± 2.1	13.2 ± 1.1	16.8 ± 1.0	17.9 ± 1.9	*p* < 0.001	*p* = 0.590	*p* = 0.170
Midpoint exercise test, min	14.0 ± 2.4	15.7 ± 2.0	17.2 ± 1.0	20.1 ± 2.8	*p* < 0.001	*p* = 0.006	*p* = 0.432
Final exercise test, min	14.8 ± 2.1	20.4 ± 2.2	17.2 ± 0.9	25.0 ± 6.5	*p* = 0.011	*p* < 0.001	*p* = 0.386

*Note*: Data are presented as mean ± standard deviation.

Abbreviations: SF, sedentary female; SM, sedentary male; TF, trained female; TM, trained male.

^#^ *p* < 0.05 versus SM.

^†^ p < 0.05 versus TM.

### 3.2 Maximal exercise capacity

Performance in the treadmill test, measured in minutes, revealed a main effect of sex at baseline (*p* < 0.001), mid‐intervention (p < 0.001), and post‐intervention (after 8 weeks of placebo or exercise training) (*p* < 0.001), with male rats exhibiting lower performance compared to females. Additionally, a main effect of exercise training was observed at the mid‐ (*p* = 0.006) and post‐intervention (*p* < 0.001) time points, indicating improved endurance in trained male and female animals (Table 1).

### 3.3 Cardiovascular parameters

Data on hemodynamic parameters and baroreflex sensitivity are summarized in Table 2. A significant main effect of sex was observed for SAP (*p* = 0.033), DAP (*p* = 0.005), and MAP (p = 0.005) after 8 weeks of placebo or intervention, with male rats exhibiting higher values than females. In addition, a main effect of exercise training was found for HR (*p* = 0.007). Specifically, exercise training led to a reduction in HR. No significant interaction effects between sex and exercise training were observed for any of the analyzed variables.

**TABLE 2:** Hemodynamic and baroreflex sensitivity assessments in the studied groups.

	Male		Female		Two‐way ANOVA		
	SM	TM	SF	TF	Sex effect	Exercise training	Interaction
SAP, mmHg	136 ± 7	134 ± 7	132 ± 8	129 ± 7	*p* = 0.033	*p* = 0.207	*p* = 0.841
DAP, mmHg	103 ± 8	99 ± 6	96 ± 6	93 ± 9	*p* = 0.005	*p* = 0.112	*p* = 0.861
MAP, mmHg	119 ± 7	116 ± 6	113 ± 6	110 ± 8	*p* = 0.005	*p* = 0.110	*p* = 0.945
HR, bpm	351 ± 33	332 ± 21	363 ± 24	338 ± 22	*p* = 0.265	*p* = 0.007	*p* = 0.765
Alpha index, ms/mmHg	0.62 ± 0.24	0.81 ± 0.28	0.62 ± 0.30	0.94 ± 0.36	*p* = 0.536	*p* = 0.017	*p* = 0.506

*Note*: Data are presented as mean ± standard deviation.

Abbreviations: DAP, diastolic arterial pressure; HR, heart rate; MAP, mean arterial pressure; SAP, systolic arterial pressure; SF, sedentary female; SM, sedentary male; TF, trained female; TM, trained male.

### 3.4 Cardiovascular autonomic modulation

**FIGURE 1:**
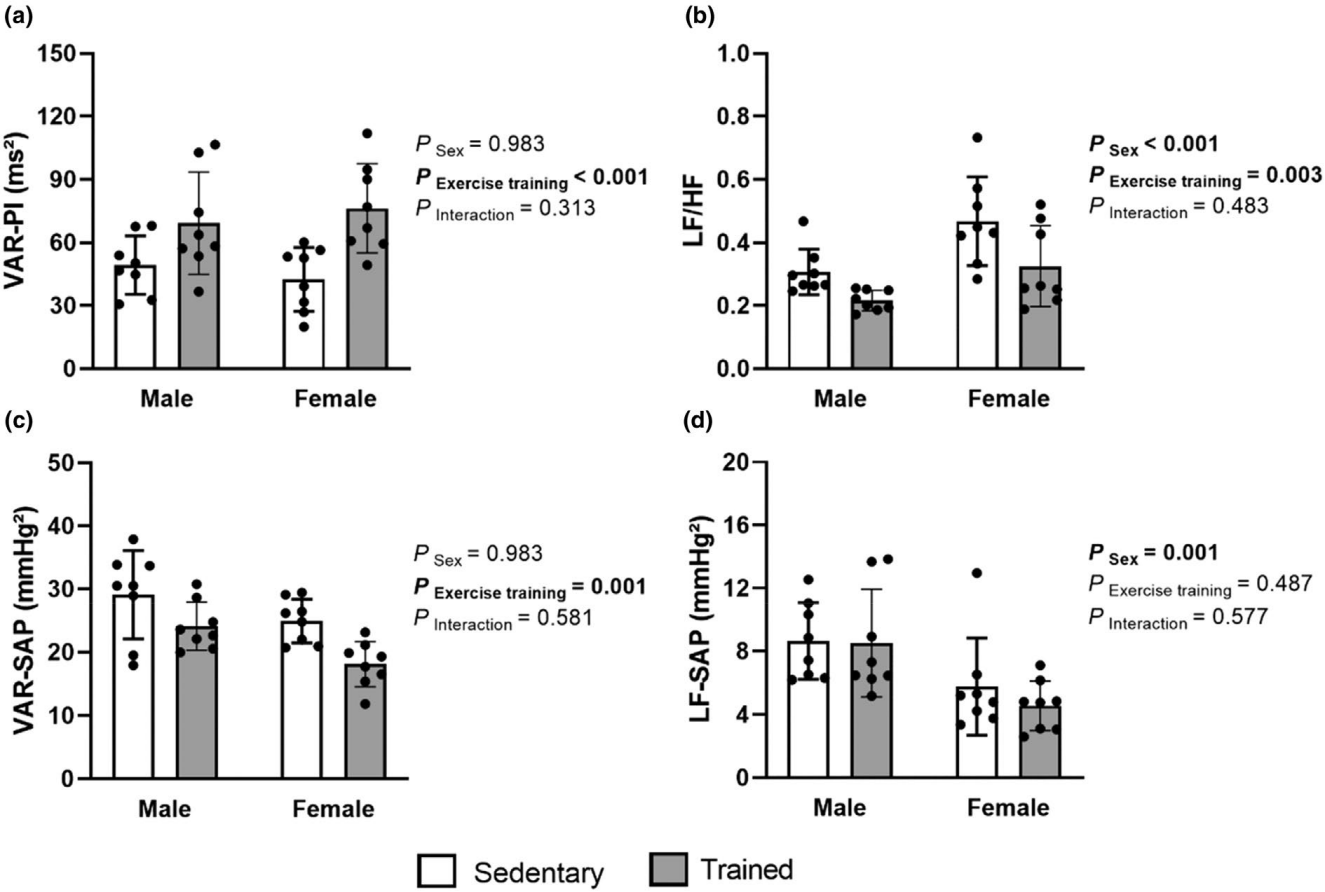
Cardiovascular autonomic control. Variance of pulse interval (VAR‐PI) (a), LF/HF ratio (b), variance of systolic arterial pressure (VAR‐SAP) (c), and low‐frequency band of systolic arterial pressure (LF‐SAP) (d). Data are presented as mean ± standard deviation. Two‐way ANOVA followed by Tukey's post hoc test, 8 rats per group. SM, sedentary male; SF, sedentary female; TF, trained female; TM, trained male.

Analysis of autonomic variables revealed distinct effects. Pulse interval variance (VAR‐PI) reveals an effect of exercise training (*p* < 0.001), with trained rats (TM: 69.1 ± 24.4 and TF: 76.2 ± 21.2 mmHg^2^) exhibiting greater variability compared to sedentary ones (SM: 49.3 ± 13.9 and SF: 42.5 ± 15.2 mmHg^2^) (Figure 1a). For the LF/HF ratio, there were significant effects of both sex (*p* < 0.001) and exercise training (*p* = 0.003). Males (SM: 0.307 ± 0.072 and TM: 0.216 ± 0.033) showed lower values compared to females (SF: 0.468 ± 0.141 and TF: 0.325 ± 0.128), and trained rats exhibited lower LF/HF ratios than sedentary counterparts (Figure 1b). Regarding variance of SAP (VAR‐SAP), a main effect of exercise training (*p* = 0.001) was observed, with trained groups (TM: 24.1 ± 3.8 and TF: 18.1 ± 3.6 mmHg^2^) showing lower values in relation to sedentary groups (SM: 29.1 ± 6.99 and SF: 25.0 ± 3.5 mmHg^2^) (Figure 1c). For the low‐frequency component of SAP (LF‐SAP), a sex effect (p = 0.001) was found, with males (SM: 8.65 ± 2.43 and TM: 8.52 ± 3.41 mmHg^2^) presenting higher values than females (SF: 5.77 ± 3.07 and TF: 4.55 ± 1.58 mmHg^2^) (Figure 1d). Furthermore, there was a main effect of exercise training on baroreflex sensitivity (*p* = 0.017), as assessed by the alpha index. Specifically, exercise training led to an increase in the alpha index, indicating improved baroreflex sensitivity (Table 2). No significant interaction between sex and training was observed for any of the parameters analyzed.

### 3.5 Nitric oxide bioavailability

A main effect of sex (*p* < 0.012) and interaction between sex and exercise training (*p* = 0.002) was observed for plasma nitrite concentrations (nmol/mg protein). Post hoc analysis revealed that sedentary females exhibited lower nitrite levels compared to sedentary males, whereas exercise training was effective in increasing these levels in females (Table 3).

**TABLE 3:** Cardiac and renal antioxidant enzyme activity in the studied groups.

	Male		Female		Sex	Exercise training	Interaction
	SM	TM	SF	TF			
Cardiac tissue							
SOD, U/mg protein	15.1 ± 1.69	14.0 ± 2.05	15.7 ± 2.69	15.5 ± 1.53	*p* = 0.173	*p* = 0.394	*p* = 0.529
CAT, nmol/mg protein	0.518 ± 0.038	0.806 ± 0.078*	0.796 ± 0.171^#^	0.677 ± 0.150	*p* = 0.122	*p* = 0.079	*p* < 0.001
GPx, nmol/mg protein	0.029 ± 0.004	0.012 ± 0.001*	0.034 ± 0.011^†^	0.035 ± 0.011^†^	*p* < 0.001	*p* = 0.030	*p* = 0.015
Renal tissue							
SOD, U/mg protein	10.8 ± 1.43	8.24 ± 2.87	10.2 ± 2.82	11.0 ± 2.90	*p* = 0.290	*p* = 0.369	*p* = 0.094
CAT, nmol/mg protein	2.12 ± 0.43	4.15 ± 1.68	1.72 ± 0.85	2.44 ± 1.05	*p* = 0.013	*p* = 0.002	*p* = 0.112
GPx, nmol/mg protein	0.061 ± 0.023	0.034 ± 0.011	0.044 ± 0.011	0.035 ± 0.014	*p* = 0.148	*p* = 0.003	*p* = 0.113
Plasma							
Nitrite, nmol/mg protein	0.847 ± 0.299	0.616 ± 0.262	0.327 ± 0.100^#^	0.680 ± 0.143*	*p* = 0.012	*p* = 0.549	*p* = 0.002

*Note*: Data are presented as mean ± standard deviation.

Abbreviations: CAT, catalase; GPx, glutathione peroxidase; SF, sedentary female; SM, sedentary male; SOD, superoxide dismutase; TF, trained female; TM, trained male.

* *p* < 0.05 versus sedentary control.

^#^ *p* < 0.05 versus SM.

^†^ *p* < 0.05 versus TM.

### 3.6 Cardiac oxidative stress

**FIGURE 2:**
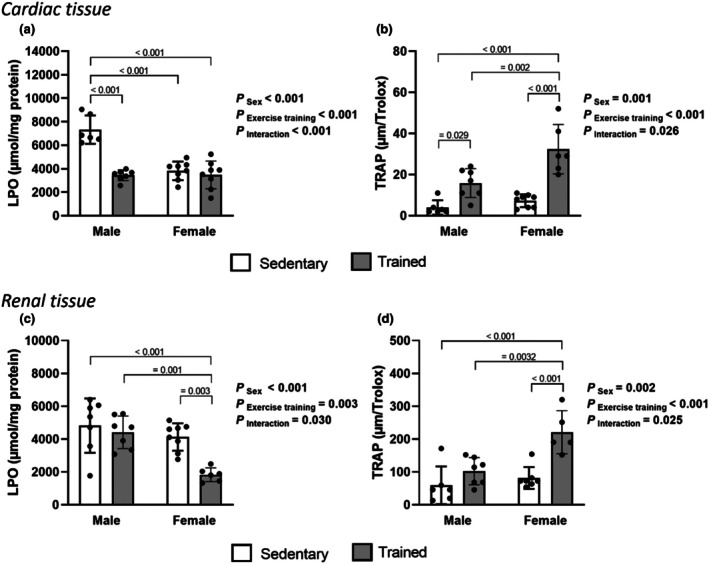
Cardiac and renal oxidative stress profile. Cardiac lipid peroxidation (a), cardiac total antioxidant capacity (TRAP) (b), renal lipid peroxidation (c), and renal total antioxidant capacity (TRAP) (d). Data are presented as mean ± standard deviation. Two‐way ANOVA followed by Tukey's post hoc test, 6–8 rats per group (TF group = 5 rats for renal TRAP). SM, sedentary male; SF, sedentary female; TF, trained female; TM, trained male.

LPO levels (μmol/mg protein) in cardiac tissue, assessed by chemiluminescence (QL), are presented in Figure 2a. A significant main effect of sex (*p* < 0.001), exercise training (p < 0.001), and interaction between sex and exercise training (p < 0.001) was detected. Post hoc analysis revealed that sedentary male rats had higher levels of LPO compared to the other groups (SM: 7323 ± 1209 vs. TM: 3442 ± 456, SF: 3815 ± 794, and TF: 3478 ± 1185). In addition, exercise training was effective in reducing LPO in male rats. Furthermore, the main effect of sex (*p* = 0.001), exercise training (*p* < 0.001), and the interaction between sex and exercise training (*p* = 0.026) were observed for TRAP (μM/Trolox) in cardiac tissue. Here, the trained female group exhibited higher TRAP values compared to other groups (TF: 32.4 ± 12.1 vs. SF: 7.4 ± 3.4, SM: 4.0 ± 3.5, and TM: 15.9 ± 6.9), indicating that exercise training was effective in enhancing antioxidant capacity in female rats (Figure 2b). No significant effects of sex, exercise training, or interaction between these factors were observed for SOD activity. In contrast, CAT activity showed an interaction between sex and exercise training (*p* < 0.001). Post hoc analysis indicated that SM exhibited lower CAT activity compared to TM and SF groups. Regarding GPx activity, there is a significant main effect of sex (p < 0.001), exercise training (*p* = 0.030), as well as an interaction between the two factors (*p* = 0.015). Post hoc analysis showed that TM presented lower GPx activity compared to SM, SF, and TF groups (Table 3).

**FIGURE 3:**
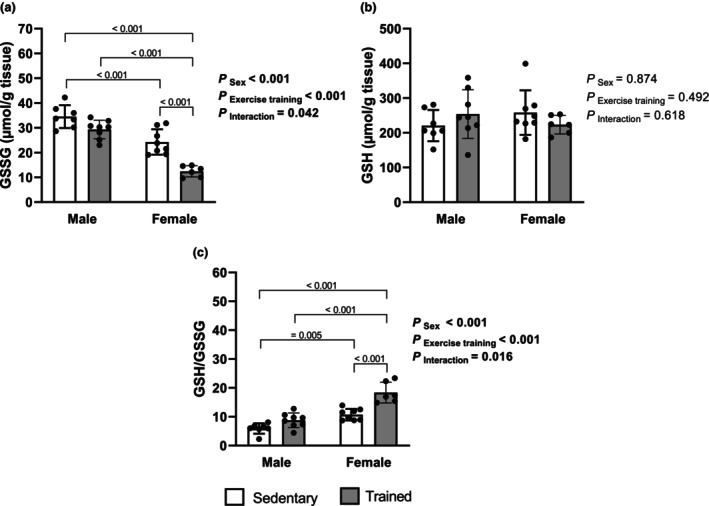
Cardisc oxidized and reduced glutathione redox balance. Oxidized glutathione form (GSSG) (a), reduced glutathione form (GSH) (b), and GSH/GSSG ratio (c). Data are presented as mean ± standard deviation. Two‐way ANOVA followed by Tukey's post hoc test, 6–8 rats per group. SF, sedentary female; SM, sedentary male; TF, trained female; TM, trained male.

Regarding GSSG levels (μmol/mg tissue), a main effect of sex (*p* < 0.001), exercise training (p < 0.001), and interaction (*p* = 0.042) was observed. Specifically, post hoc analysis revealed that trained females exhibited lower values (TF: 12.4 ± 2.3) compared to males (SM: 34.5 ± 4.6; TM: 29.3 ± 3.8), as well as sedentary females showing lower GSSG (SF: 24.3 ± 5.1) in relation to the SM group. In addition, exercise training induced a marked reduction in GSSG levels in both sexes. No significant interaction between sex and training was observed for GSSG. In the same way, no significant effect was detected for GSH levels (SM: 221 ± 45; TM: 254 ± 70; SF: 258 ± 64; and TF: 223 ± 27). However, significant effects of sex (*p* < 0.001), exercise training (*p* = 0.001), and interaction (*p* < 0.016) were observed for GSH/GSSG. Post hoc analysis revealed that trained females (TF: 18.4 ± 3.6) exhibited a higher GSH/GSSG ratio compared to other groups (SM: 5.9 ± 1.8, TM: 8.8 ± 2.5, and SF: 10.7 ± 2.0). Moreover, sedentary females showed a higher GSH/GSSG ratio compared to sedentary males (Figure 3a–c).

### 3.7 Renal oxidative stress

There is a main effect of sex (*p* < 0.001), exercise training (*p* = 0.003), and interaction between sex and exercise training (*p* = 0.030) for LPO (μmol/mg protein) in renal tissue. Post hoc showed that trained female rats (TF: 1832 ± 418) showed lower LPO levels compared to other groups (SM: 4825 ± 1652, TM: 4415 ± 989, and SF: 4132 ± 833) (Figure 2c). In addition, there was a main effect of sex (*p* = 0.002), exercise training (*p* < 0.001), and interaction between sex and exercise training (*p* = 0.025) for TRAP (μM/Trolox) in renal tissue. Post hoc analysis showed that exercise training increased TRAP levels in female rats, with trained females (TF: 221.0 ± 65.9) displaying higher antioxidant capacity compared to both sedentary females (SF: 81.6 ± 33.2) and male groups (SM: 59.3 ± 57.6 and TM: 102.0 ± 41.5) (Figure 2d). On the other hand, no significant effects were observed for SOD activity. In contrast, a significant main effect of sex was found for CAT activity (*p* = 0.013), with female rats showing lower CAT levels compared to males. Additionally, a main effect of exercise training was detected (*p* = 0.002), with trained animals exhibiting higher CAT activity compared to sedentary groups. In turn, a significant main effect of exercise training was observed for GPx activity (*p* = 0.003), with trained rats exhibiting lower GPx activity compared to sedentary groups (Table 3).

**FIGURE 4:**
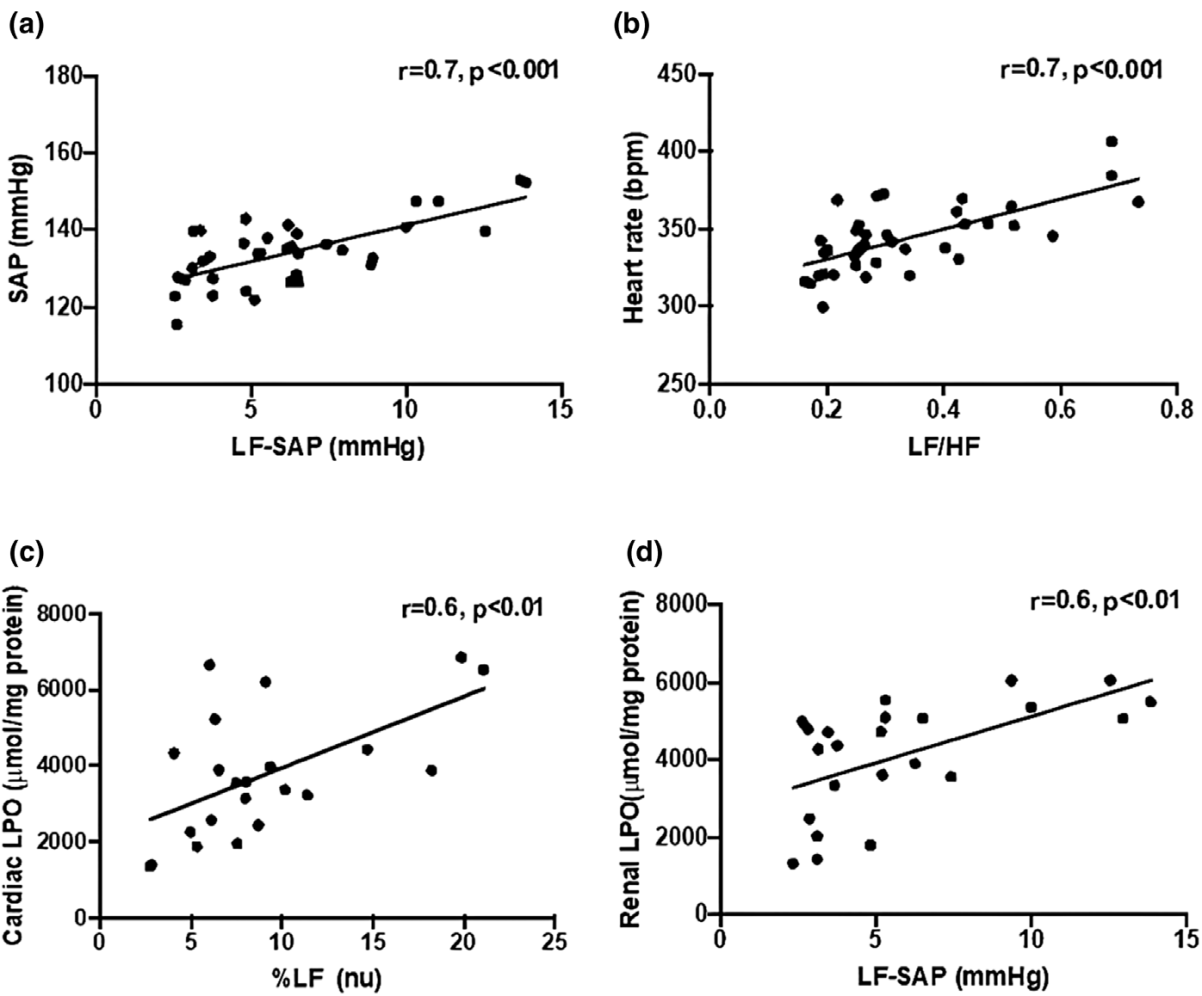
Relationship between cardiovascular autonomic modulation and basal hemodynamics and oxidative damage in target organs. Systolic arterial pressure (SAP) and low‐frequency band of systolic arterial pressure (LF‐SAP) (a), heart rate (HR) and cardiac low frequency band/high frequency band of pulse interval (LF/HF) ratio (b), low frequency band of pulse interval (LF) and cardiac lipid peroxidation (LPO) (c), and low‐frequency band of systolic arterial pressure (LF‐SAP) and renal lipid peroxidation (LPO) (d). Pearson correlation coefficient.

Positive correlations were found between LF‐SAP and SAP (*r* = 0.7, *p* < 0.001) (Figure 4a), and between the LF/HF ratio and HR (*r* = −0.6, *p* < 0.001) (Figure 4b). In addition, positive correlations were observed between the LF band of PI and cardiac LPO (*r* = 0.6, *p* < 0.01) (Figure 4c) and between LF‐SAP and renal LPO (*r* = 0.6, *p* < 0.01) (Figure 4d).

## 4 DISCUSSION

Assessing sex differences is crucial for understanding variations in cardiovascular risk, as males and females exhibit distinct biological and hormonal profiles that influence disease pathophysiology. Evaluating responses to exercise training is also essential to determine how these sex‐specific physiological characteristics affect adaptation to exercise. Such insights support the development of more effective, personalized interventions aimed at improving cardiovascular health and reducing disease risk.

The present study investigated sex differences in cardiovascular autonomic modulation and oxidative stress in target organs under baseline conditions and following 8 weeks of aerobic exercise training. The main findings reveal distinct sex‐related variations in anthropometric, functional, autonomic, and oxidative stress parameters. Furthermore, aerobic exercise training elicited significant improvements in functional, cardiovascular autonomic regulation, and oxidative stress profile. However, these adaptations were more pronounced in the female group. This sex‐specific adaptation suggests that females may exhibit greater sensitivity or adaptability to aerobic training, as evidenced by more pronounced improvements in lipid peroxidation and antioxidant capacity in renal tissue, as well as GSSG and the GSH/GSSG ratio. These findings reinforce the importance of considering sex as a critical biological variable that influences exercise‐induced physiological adaptations.

Compared to the sedentary groups, the trained female exhibited approximately 269% higher renal TRAP and ~49% lower cardiac GSSG levels. In contrast, the trained male showed a 172% increase in renal TRAP and a 15% reduction in cardiac GSSG. These results indicate that trained females demonstrated a greater magnitude of adaptation in terms of renal TRAP (~1.6 times higher) and cardiac GSSG (~3.3 times lower) compared to trained males. Furthermore, although both trained males and females showed similar reductions in cardiac LPO, only the female rats exhibited a reduction in renal LPO, an increase in both antioxidant capacity and an improvement in cardiac glutathione redox balance in response to aerobic exercise training. These findings suggest that females exhibit more efficient oxidative stress adaptations than males. Overall, the data support the hypothesis that biological sex is a critical variable influencing the magnitude of physiological responses to aerobic training, particularly under conditions of oxidative stress.

It is well established that sex‐based differences exist in exercise performance, with males typically demonstrating greater aerobic capacity and muscular strength than females (Hunter & Senefeld, 2024). However, such differences are not always consistently observed in rodent models. In our study, female rats exhibited higher exercise performance than their male counterparts. Previous findings from our group have shown that performance gains following aerobic exercise training are comparable between sexes (Silva et al., 2025). In the present study, 8 weeks of aerobic training led to increased exercise capacity in both male and female rats. We propose that anthropometric factors may partially account for the similar performance outcomes observed between sexes in experimental models. Female rats generally have lower body weight than age‐matched males, a pattern also observed in our study. Given that maximal oxygen consumption is influenced by body mass, the lower body weight of females may result in a reduced relative workload per kilogram during treadmill running. This could, in turn, enhance endurance performance during the exercise test.

The autonomic nervous system is a key regulator of cardiovascular homeostasis (Venkata Pothineni et al., 2016). There are multiple ways to measure cardiac autonomic control. HR and APV are used to assess the role of autonomic nervous system fluctuations in various cardiovascular and non‐cardiovascular disorders (Pagani, 1997). Recent data suggest that estrogen provides a degree of cardiovascular protection through its influence on the autonomic nervous system (Dutra et al., 2013; Venkata Pothineni et al., 2016). Notably, we have demonstrated that the ovulatory phase and ovarian hormone deprivation induce an increase in AP compared to the non‐ovulatory phase (Ferreira et al., 2020). These conditions reflect impaired baroreflex sensitivity and are associated with higher APV (Ferreira et al., 2020). Previous studies have shown that healthy premenopausal women exhibit lower cardiac sympathetic modulation compared to men (Kuo et al., 1999). This finding is consistent with the lower LF‐SAP observed in female groups in the present study. These data could help to explain the lower cardiovascular risk in premenopausal women compared to men in the early stages of life. Additionally, aerobic exercise training improved HR variability and sympathovagal balance (LF/HF), independently of sex, which was reflected in a reduction in resting heart rate. Indeed, the cardiac benefits induced by aerobic exercise training appear to be independent of sex, despite differences in cardiovascular autonomic modulation in sedentary conditions (Dutra et al., 2013).

In our study, female rats exhibited lower AP compared to males, a finding that aligns with the reduced LF‐SAP observed in this group, suggesting lower sympathetic modulation of vascular tone. On the other hand, studies have shown that aerobic exercise training does not affect AP in either male (De Angelis et al., 1997) or female (Sanches et al., 2009) normotensive rats. In the present study, AP values obtained did not change after aerobic exercise training in either sex. However, baroreflex sensitivity, as measured by the alpha index, was higher in the trained groups. This suggests that aerobic exercise training has a similar effect on baroreflex sensitivity in both sexes. The absence of sex differences under the same exercise training conditions may reflect physiological convergence in baroreceptor responsiveness elicited by chronic exercise training. In contrast, female animals also showed lower values in the index of vascular sympathetic modulation than males, as verified in LF‐SAP. This may explain reduced vasoconstriction and lower vascular resistance in females compared to males, especially after exercise (Bassareo & Crisafulli, 2020). Interestingly, we observed a positive correlation between LF‐SAP and SAP, showing that despite similar baseline AP values in both sexes and training conditions, animals with lower sympathetic vascular modulation exhibited reduced SAP values. These data reinforce the link between autonomic dysregulation and haemodynamic alterations. Notably, we observed resting bradycardia in trained male and female rats compared to sedentary groups, which may be associated with lower cardiac sympathovagal balance. Supporting this hypothesis, we obtained moderate correlations between HR and the LF/HF ratio (*r* = 0.7) across all groups, demonstrating that animals with a lower cardiac sympathovagal balance exhibited a reduced basal HR.

Assessing oxidative stress in cardiac and renal tissues helps to detect cellular damage caused by reactive oxygen species, which are closely linked to cardiovascular and renal dysfunction. Furthermore, this analysis enables the investigation of the protective effects of exercise training, as well as potential sex‐related differences in oxidative stress responses. This contributes to the development of more effective and personalized preventive and therapeutic strategies. A relationship has been reported between decreased sympathetic modulation and enhanced central and peripheral oxidative stress (Bertagnolli et al., 2006; Irigoyen et al., 2005). Oxidative stress can occur through increased formation of reactive oxygen species and has been implicated in a large number of diseases, such as CVDs (Kander et al., 2017; Liguori et al., 2018). Furthermore, accumulating evidence indicates an estrogenic protective role in cardiovascular disorders (Flues et al., 2010; Iorga et al., 2017). In our study, sedentary female rats demonstrated lower levels of cardiac tissue membrane lipid peroxidation, as measured by CL, compared to sedentary male rats. Indeed, estrogen appears to act centrally, reducing AP, sympathoexcitation and oxidative stress (Hao et al., 2016).

Nicotinamide adenine dinucleotide phosphate (NADPH) oxidase enzymes are well‐established sources of reactive oxygen species in both cardiac and renal tissues (Arendshorst et al., 2024). Sex has been shown to influence oxidative stress levels (Kander et al., 2017), with females generally exhibiting lower susceptibility under physiological conditions. This difference may be attributed to the antioxidant effects of estrogen, variations in NADPH oxidase activity between sexes, or other mechanisms that remain to be fully elucidated. Experimental (Barp et al., 2002) and clinical (Ide et al., 2002) studies have demonstrated that males exhibit greater oxidative stress than females. About this, evidence from previous studies indicates that female rats exhibit significantly lower NADPH oxidase‐derived superoxide production in cerebral arteries compared to males—with reductions reaching nearly 50% (Miller et al., 2007). These sex‐related differences are estrogen‐dependent and are associated with reduced expression of Nox1 and Nox4 in females (Miller et al., 2007). This aligns with evidence showing that Nox4 expression is markedly reduced in the mesenteric arteries of female rats relative to their male counterparts (Zhang et al., 2012). In spontaneously hypertensive rats, males generally show higher levels of oxidative stress than females, a disparity attributed to increased NADPH oxidase activity (Sartori‐Valinotti et al., 2007). In addition, nitric oxide, a molecule that plays a crucial role in various physiological processes, including vascular tone regulation, and whose bioavailability can be significantly reduced under oxidative stress, particularly through its rapid reaction with superoxide anion, is found at higher levels in females compared to males, suggesting a sex‐related advantage in nitric oxide‐mediated vascular function (Knot et al., 1999). Unfortunately, we did not measure NADPH oxidase activity or expression in our study, which limits a more detailed understanding of its contribution to the oxidative stress observed. However, we found that trained female rats exhibited increased nitrite concentrations following exercise training, whereas male rats showed a tendency toward reduced levels. This sex‐dependent response may be partially explained by the influence of estrogen in females, which is known to enhance endothelial nitric oxide synthase expression and activity, thereby promoting nitric oxide production. In contrast, males may exhibit a different regulatory profile, potentially involving higher oxidative stress or altered nitric oxide metabolism.

Exercise training decreased membrane LPO in cardiac (male) and renal tissues (female) in both trained groups. In addition, the female trained group exhibited lower levels of membrane LPO in renal tissue. These results reinforce those showing that the adaptation to oxidative stress induced by exercise training depends on tissue type (Balci et al., 2012). Consistent with the present findings, previous data from our group revealed low levels of cardiac oxidative stress markers following aerobic exercise training in ovariectomised female rats (Irigoyen et al., 2005) and male hypertensive rats (Bertagnolli et al., 2008).

Some studies have reported an important role for sex differences in antioxidant capacity and exercise‐induced reactive oxygen species production (Goldfarb et al., 2007; Yamamoto et al., 2002). However, other evidence claims that sex does not affect oxidative stress parameters after exercise training (Pepe et al., 2009), highlighting the need for further research to clarify these conflicting findings. In the present study, trained female rats also showed a marked improvement in cardiac glutathione redox balance (GSH/GSSG). This is particularly noteworthy given the pivotal role of glutathione, a major nonenzymatic endogenous antioxidant, in neutralizing reactive oxygen species and protecting tissues from oxidative damage (Hanschmann et al., 2013).

Chronic oxidative stress contributes to the development of endothelial dysfunction, highlighting the critical role of increased reactive oxygen species production in the progression of this functional impairment. It has been suggested that females possess a greater antioxidant capacity compared to males (Bhatia et al., 2012), indicating an apparent association between sex and oxidative stress, with females appearing to be less susceptible to oxidative damage. Higher SOD activity levels were observed in the brain and lungs of female mice, whereas no significant differences were found between males and females in the kidney or heart (Chen et al., 2011). In addition, castration significantly reduced SOD activity in both sexes, suggesting a role for sex hormones in regulating this enzyme (Barp et al., 2002). Overall, the relationship between SOD activity and biological sex remains unclear due to these conflicting findings (Brandes & Mügge, 1997). In turn, CAT activity was similar between sexes in the brain, heart, and lung, but higher in the female kidney (Chen et al., 2011), while other studies found no significant sex differences in CAT activity (Barp et al., 2002; Gómez‐Pérez et al., 2011). Moreover, lower GPx activity has been reported in females compared to males (Barp et al., 2002; Gómez‐Pérez et al., 2011), and castration did not significantly affect GPx activity levels, suggesting that this enzyme may not be regulated by sex hormones (Barp et al., 2002). In our study, male rats showed lower total antioxidant capacity after exercise training and higher levels of GSSG compared to females. These findings support the notion that males exhibit a less efficient redox balance, reinforcing the idea of sex‐related differences in oxidative stress regulation. Furthermore, trained male rats exhibited lower GPx activity in cardiac tissue compared to females. This difference may reflect a reduced capacity in males to neutralize hydrogen peroxide through the glutathione system. Such a response could be linked to sex‐related differences in antioxidant regulation, possibly influenced by intrinsic factors other than sex hormones, given that GPx activity appears to be less sensitive to hormonal modulation.

Previous studies have consistently shown that exercise is a powerful tool in promoting greater nitric oxide bioavailability and, consequently, improving endothelial function (Delbin et al., 2011; Dyakova et al., 2015; Tsukiyama et al., 2017), and these improvements have been directly associated with reductions in AP (Braga et al., 2015). Although no differences in nitrite levels were observed between females and males at baseline, only females showed a significant increase in this marker following exercise training. This finding suggests a greater responsiveness of the nitric oxide‐dependent system in females in response to exercise stimuli. Additionally, the concurrent increase in TRAP in this group may have contributed to enhanced nitric oxide bioavailability by reducing its degradation by reactive oxygen species. We hypothesized that these adaptations may be related to sex‐specific differences in redox signaling, the sensitivity of enzymatic pathways involved in nitric oxide synthesis—such as endothelial nitric oxide synthase—and/or the hormonal profile, particularly the vasoprotective effects of estrogen. Indeed, the superior antioxidant profile observed in trained females in both cardiac and renal tissues could be attributed to female sex hormones. These hormones are known to stimulate the antioxidant defense system by inducing the expression of antioxidant enzymes (Katalinic et al., 2005), inhibiting LPO in certain tissues (Huh et al., 1994), and ultimately enhancing mitochondrial and systemic antioxidant capacity (Borrás et al., 2003).

Notably, changes in AP or HR variability were correlated with end organ damage in both experimental and clinical evidence, despite normal AP values (Flues et al., 2012; Irigoyen et al., 2016; Malik et al., 1996). Furthermore, it is well known that oxidative stress is one of the key mechanisms involved in end organ damage that can develop during the lifespan (Liguori et al., 2018). In this sense, our data provide evidence that changes in cardiovascular autonomic modulation related to sex or intervention (e.g., exercise training) can affect oxidative stress markers in the target organ. We observed significant positive correlations between the LF band of PI and cardiac LPO. Additionally, a similar relationship was observed between LF‐SAP and renal LPO. Together, these results suggest that animals with lower cardiac and indirect indices of vascular sympathetic modulation of SAP present reduced levels of cardiac and renal oxidative damage, respectively. Specifically, a heightened indirect index of vascular sympathetic modulation may contribute to renal oxidative stress, possibly through mechanisms involving reduced perfusion and increased reactive oxygen species production.

The lack of assessment of related redox pathways, such as NADPH oxidase, can be considered a limitation, since these pathways play a crucial role in redox balance and oxidative stress modulation in target organs. Future studies may reinforce and further substantiate the present findings, contributing to a deeper understanding and confirmation of sex differences and the effects of exercise training.

In summary, the aerobic exercise training protocol improved cardiovascular autonomic modulation and the oxidative stress profile of the target organs in both sexes. Favorable changes in AP and HR variability were associated with hemodynamic improvement and reduced oxidative damage in cardiac and renal tissue. The benefits of exercise training were more pronounced in females than in males. Therefore, the findings of the present study suggest that sex is a key factor in the changes induced by aerobic exercise training. Our data reinforce the importance of considering sex when investigating cardiovascular dysfunction related to autonomic modulation and oxidative stress, and when developing approaches to prevent and treat CDVs.

## FUNDING INFORMATION

São Paulo Research Foundation (FAPESP) (n° 2007/57595‐5; 2018/17183‐4; 2019/06277‐0), and National Council for Scientific and Technological Development (CNPq: 151838/2024‐0, 407398/2021‐0, 406792/2022‐4, and 408899/2024‐7). M.J.F. (CNPq—PDJ), K.D.A and M.C.I. (CNPq—BPQ) are recipients of CNPq Fellowship.

## CONFLICT OF INTEREST STATEMENT

The authors declare that they have no competing interests.

## ETHICS STATEMENT

Ethics Committee of São Judas Tadeu University (protocol n. 076/2004).

## Data Availability

The datasets used and/or analyzed during the current study are available from the corresponding author on reasonable request.
